# Poor outcome of allogeneic transplantation for therapy-related acute myeloid leukemia induced by prior chemoradiotherapy

**DOI:** 10.1007/s00277-023-05356-6

**Published:** 2023-07-21

**Authors:** Hiroaki Araie, Yasuyuki Arai, Michiko Kida, Jun Aoki, Naoyuki Uchida, Noriko Doki, Takahiro Fukuda, Masatsugu Tanaka, Yukiyasu Ozawa, Masashi Sawa, Yuta Katayama, Yayoi Matsuo, Makoto Onizuka, Yoshinobu Kanda, Toshiro Kawakita, Junya Kanda, Yoshiko Atsuta, Masamitsu Yanada, for the Adult Acute Myeloid Leukemia Working Group of the Japanese Society for Transplantation and Cellular Therapy

**Affiliations:** 1 Department of Hematology and Oncology, Faculty of Medical Sciences, University of Fukui, 23-3 Matsuoka Shimoaizuki, Eiheiji-cho, Yoshida-gun, Fukui, 910-1193 Japan; 2 Department of Hematology and Oncology, Graduate School of Medicine, Kyoto University, Kyoto, Japan; 3 Department of Hematology, NTT Medical Center Tokyo, Tokyo, Japan; 4 Department of Hematopoietic Stem Cell Transplantation, National Cancer Center Hospital, Tokyo, Japan; 5 Department of Hematology, Federation of National Public Service Personnel Mutual Aid Associations TORANOMON HOSPITAL, Tokyo, Japan; 6 Hematology Division, Tokyo Metropolitan Cancer and Infectious Diseases Center, Komagome Hospital, Tokyo, Japan; 7 Department of Hematology, Kanagawa Cancer Center, Yokohama, Japan; 8 Department of Hematology, Japanese Red Cross Aichi Medical Center Nagoya Daiichi Hospital, Aichi, Japan; 9 Department of Hematology and Oncology, Anjo Kosei Hospital, Aichi, Japan; 10 Department of Hematology, Hiroshima Red Cross Hospital & Atomic-bomb Survivors Hospital, Hiroshima, Japan; 11 Department of Hematology, Hamanomachi Hospital, Fukuoka, Japan; 12 Department of Hematology/Oncology, Tokai University School of Medicine, Isehara, Japan; 13 Division of Hematology, Jichi Medical University Saitama Medical Center, Saitama, Japan; 14 Department of Hematology, National Hospital Organization Kumamoto Medical Center, Kumamoto, Japan; 15 Japanese Data Center for Hematopoietic Cell Transplantation, Nagakute, Japan; 16 Department of Registry Science for Transplant and Cellular Therapy, Aichi Medical University School of Medicine, Nagakute, Japan; 17 Department of Hematology and Cell Therapy, Aichi Cancer Center, Nagoya, Japan

**Keywords:** Therapy-related acute myeloid leukemia, Allogeneic hematopoietic stem cell transplantation, Secondary malignancy, Treatment outcome

## Abstract

Therapy-related acute myeloid leukemia (t-AML) is a therapeutic challenge as a late complication of chemotherapy (CHT) and/or radiotherapy (RT) for primary malignancy. Allogeneic hematopoietic stem cell transplantation (allo-HSCT) presents itself as a curative approach. To establish the optimal allo-HSCT strategy for t-AML, we evaluated the relationship between characteristics of primary malignancy and allo-HSCT outcomes. Patients with t-AML or de novo acute myeloid leukemia (AML) who underwent first allo-HSCT in Japan from 2011 to 2018 were identified using a nationwide database. The detailed background of t-AML was obtained by additional questionnaires. Multivariate analysis and propensity score matching (PSM) analysis were performed to detect the prognostic factors associated with t-AML and compare outcomes with de novo AML. We analyzed 285 t-AML and 6761 de novo AML patients. In patients with t-AML, receiving both CHT and RT for primary malignancy was an independent poor-risk factor for relapse and overall survival (OS) (hazard ratio (HR) 1.62; *p* = 0.029 and HR 1.65; *p* = 0.009, reference: CHT alone group), whereas other primary malignancy-related factors had no effect on the outcome. Compared to the CHT alone group, complex karyotypes were significantly increased in the CHT + RT group (86.1% vs. 57.5%, *p* = 0.007). In the PSM cohort, t-AML patients with prior CHT and RT had significantly worse 3-year OS than those with de novo AML (25.2% and 42.7%; *p* = 0.009). Our results suggest that prior CHT and RT for primary malignancy may be associated with increased relapse and worse OS of allo-HSCT in t-AML.

**Supplementary Information:**

The online version contains supplementary material available at 10.1007/s00277-023-05356-6.

## Introduction

Therapy-related acute myeloid leukemia (t-AML) is an important category of myeloid malignancies. The World Health Organization (WHO) classification defines t-AML as acute myeloid leukemia (AML) that develops as a late complication of cytotoxic chemotherapy (CHT) and/or radiation therapy (RT) for malignant or non-malignant disease [1, 2]. While therapeutic advances have improved survival for many cancer patients, t-AML cases are increasing annually [3, 4] and account for 6–8% of all AML patients [5–7].

Compared to de novo AML, t-AML patients have a dismal prognosis because of their older age, higher proportion of unfavorable cytogenetic abnormalities, and cumulative toxicity of therapy for the primary malignancy [5, 6, 8, 9]. As a curative approach, allogeneic hematopoietic stem cell transplantation (allo-HSCT) has been performed with t-AML patients who had poor prognostic factors, resulting in improved outcomes compared to patients who did not undergo allo-HSCT [10–13].

As with de novo AML, prognostic factors such as age, performance status, disease status, and type of cytogenetic abnormality influence the outcome of allo-HSCT for patients with t-AML [14–16]. In addition, relapse and non-relapse mortality (NRM) after allo-HSCT may be increased in t-AML, even if the patient’s background is similar to one with de novo AML [5, 8]. However, patients with t-AML are a highly heterogeneous group because the primary malignancy and its treatment vary widely [3, 5, 15]. Although differences in the primary malignancy and its therapeutic approach may affect relapse and NRM, the relationship between the background of the primary malignancy and the outcome of allo-HSCT for t-AML remains unclear.

More accurate assessment of the risk of allo-HSCT for patients with t-AML is important to support their decision-making and provide adequate supportive care. Our study objectives were to evaluate the influence of primary malignancy and its therapeutic approach on the outcome of allo-HSCT for t-AML in a nationwide registry data combined with additional research.

## Patients and methods

### Patients

For this retrospective cohort study, patient, disease, and transplantation characteristics were obtained from the nationwide registration data of the Japanese Society for Transplantation and Cellular Therapy (JSTCT) and the Japanese Data Center for Hematopoietic Cell Transplantation (JDCHCT). We included patients ≥ 16 years old with t-AML and de novo AML who underwent their first allo-HSCT from 2011 to 2018. Among t-AML patients, an additional national survey was conducted in each participating institution to obtain clinical information regarding the primary malignancy and its treatment. This study was conducted according to the Declaration of Helsinki and approved by the data management committees of the Transplant Registry Unified Management Program (TRUMP) [17, 18] and by the Institutional Review Board of The University of Fukui School of Medical Sciences. All patients provided written informed consent for collecting their data in the TRUMP.

### Definition and endpoints

t-AML was defined as patients who received CHT and/or RT for primary malignancy with AML occurring as a late complication [1, 2]. In this study, t-AML patients who underwent CHT for non-malignant disease were excluded because this study aimed to evaluate the relationship between the outcome of allo-HSCT and primary malignancy. De novo AML was defined as the diagnosis of acute myeloid leukemia with recurrent genetic abnormalities, acute myeloid leukemia with myelodysplasia-related change without the antecedent myelodysplastic syndrome, and acute myeloid leukemia not otherwise specified, according to WHO diagnostic criteria [2]. The cytogenetic risk for t-AML and de novo AML was defined according to the criteria specified by the National Comprehensive Cancer Network Guidelines [19]. The disease status at allo-HSCT was classified as first, second, third, or more complete remission (CR1, CR2, or CR3-) or non-remission (NR). The intensity of the conditioning regimens was classified as myeloablative conditioning (MAC) or reduced-intensity conditioning (RIC) according to established criteria [20]. Chemotherapy agents for primary malignancy were categorized as alkylator agent (AA), including nitrogen mustard, alkylsulfonate, nitrosourea, triazene, and platinum, and topoisomerase II inhibitor (TI), including epipodophyllotoxin and anthracycline, and others.

The endpoints were overall survival (OS), disease free survival (DFS), relapse incidence (RI), non-relapse mortality (NRM), and graft-versus-host disease (GVHD). OS was defined as the time from transplantation to death due to any cause, DFS was the time from transplantation to relapse or death due to any cause, RI as the time from transplantation to relapse of AML, and NRM as the time from transplantation to death due to any cause in remission. Acute and chronic GVHD were assessed according to standard criteria [21, 22].

### Statistical analyses

The analyses were conducted as follows: first, an assessment of patient characteristics with respect to t-AML; second, univariate and multivariate analysis of transplant outcomes using a cause-specific hazard function for OS and a cumulative incidence function for RI and NRM; third, a comparison of patient characteristics between t-AML and de novo AML; fourth, univariate and multivariate analysis of outcomes including all t-AML and de novo AML patients; fifth, validation of the above analyses using propensity score matching (PSM) in both t-AML and de novo AML cases. Patient characteristics were compared in t-AML, and between t-AML and de novo AML using the Fisher’s exact test for categorical variables and the Mann–Whitney *U* test for continuous variables. OS and DFS were estimated by the Kaplan–Meier method and was compared using the log-rank test. To identify risk factors associated with OS, univariable and multivariable Cox proportional-hazard regression models were used. Gray’s method was used to estimate the probabilities of RI and NRM, and acute and chronic GVHD. Competing events were death without relapse for RI, relapse for NRM, and death without GVHD for acute and chronic GVHD. The Fine–Gray proportional-hazard model was used to identify risk factors associated with RI and NRM. The variables to be considered were age (divided by median), sex, Eastern Cooperative Oncology Group Performance Status (ECOG-PS) (0–1 vs. 2–4), hematopoietic cell transplantation-specific comorbidity index (HCT-CI) (0 vs. 1-2 vs. 3-), cytogenetic risk (favorable vs. intermediate vs. poor), disease risk at transplantation (low risk: CR1 and CR2 vs. high risk: CR3- and NR), donor source (related bone marrow (Rel-BM) vs. related peripheral blood (Rel-PB) vs. unrelated bone marrow (UR-BM) vs. unrelated peripheral blood (UR-PB) vs. cord blood (CB)), and conditioning (MAC vs. RIC). In t-AML patients, the type of primary malignancy (solid tumor vs. hematological malignancy), treatment (CHT alone vs. RT alone vs. CHT and RT), type of chemotherapy agent (AA-based vs. TI-based vs. AA and TI-based vs. others vs. non CHT (RT alone)), and autologous peripheral blood stem cell transplantation (auto-PBSCT) were also considered as variables. If a variable contained more than 5% missing values, missing data were categorized as a separate item.

The variables included in the final multivariable models were age, sex, ECOG-PS, HCT-CI, cytogenetic risk, disease risk, donor source, and conditioning for OS; cytogenetic risk, disease risk, donor source, and conditioning for RI; and age, sex, ECOG-PS, HCT-CI, donor source, and conditioning for NRM. In addition, the factor associated with t-AML with a *p*-value of < 0.1 in each univariable analysis was also included in each multivariable model, and hazard ratios with 95% confidence intervals (CI) were calculated. Two-sided *p*-values of < 0.05 were considered statistically significant. The potential interactions between the outcome and selected significant risk factors in the multivariable Cox model were also tested by adding interaction terms to the model. PSM was performed at a one-to-one ratio using a nearest-neighbor matching method with a caliper width of 0.2 of the standard deviation of the logit of the propensity score. The final cohort was evaluated by the Mantel–Haenszel test and histogram to confirm adequate matching. All statistical analyses were performed with EZR version 1.55 (Saitama Medical Center, Jichi Medical University, Saitama, Japan), which is a graphical user interface for R (The R Foundation for Statistical Computing, Vienna, Austria, version 4.1.2). More precisely, it is a modified version of R commander (version 2.7-1) designed to add statistical functions frequently used in biostatistics [23].

## Results

### t-AML patient characteristics

We obtained information on primary malignancy and its treatment from registry data and the additional nationwide survey. Finally, a total of 285 t-AML patients were included. The characteristics of patients with t-AML are presented in Table 1 and Supplemental Table 1. The median age was 57 years, with 40% of the population over 60 years old. Female sex, HCT-CI ≥ 3, poor cytogenetic risk, high disease risk, CB, and RIC were more frequent. HCT-CI included a history of solid tumors; thus, scores tended to be high among t-AML patients. The most frequent primary malignancy was breast cancer among solid tumors and malignant lymphoma among hematologic malignancies (26.3% and 25.3%, respectively). CHT alone was the most frequent treatment for primary malignancies (67.4%), followed by CHT + RT (27%) with RT alone being the least frequent (5.6%). Among patients who received chemotherapy, more than half received combined AA and TI-based regimens (55.5%), whereas AA-based and TI-based regimens accounted for 22.8% and 8.4%, respectively. The radiation site of RT was the primary lesion in 69.8% of the CHT + RT group. The mean total radiation dose administered was 50 Gy with a standard deviation of 15.4, and the dose range was 4 to 109.5 Gy in the CHT + RT group (Supplemental Table 2). Patients who underwent auto-PBSCT for primary malignancies were 25 (8.8%): 23 with lymphoid malignancy and 2 with breast cancer. The median period from therapy to the diagnosis of t-AML was 4.5 years. The median follow-up period of survivors was 3.4 years.

**Table 1:** Patient characteristics of t-AML

	All patients	Treatment for primary malignancy		
		CHT alone	RT alone	CHT + RT
No. of patients, *N* (%)	285 (100)	192 (100)	16 (100)	77 (100)
Age				
Median [range]	57 [16–80]	58.00 [17.00, 80.00]	57.50 [30.00, 68.00]	51.00 [16.00, 80.00]
16–54	120 (42.1)	71 (37.0)	6 (37.5)	43 (55.8)
≧ 55	165 (57.9)	121 (63.0)	10 (62.5)	34 (44.2)
Sex				
Female	166 (58.2)	103 (53.6)	10 (62.5)	53 (68.8)
Male	119 (41.8)	89 (46.4)	6 (37.5)	24 (31.2)
ECOG-PS				
0–1	267 (93.7)	177 (92.2)	16 (100.0)	74 (96.1)
2–4	18 (6.3)	15 (7.8)	0 (0.0)	3 (3.9)
HCT-CI				
0	52 (18.2)	44 (22.9)	0 (0.0)	8 (10.4)
1–2	20 (7.0)	19 (9.9)	0 (0.0)	1 (1.3)
3-	208 (73.0)	125 (65.1)	16 (100.0)	67 (87.0)
Unknown	5 (1.8)	4 (2.1)	0 (0.0)	1 (1.3)
FAB classification				
M0	21 (7.4)	16 (8.3)	2 (12.5)	3 (3.9)
M1	30 (10.5)	26 (13.5)	1 (6.2)	3 (3.9)
M2	102 (35.8)	61 (31.8)	6 (37.5)	35 (45.5)
M3	7 (2.5)	6 (3.1)	0 (0.0)	1 (1.3)
M4	36 (12.6)	19 (9.9)	3 (18.8)	14 (18.2)
M5	21(7.4)	15(7.9)	1 (6.2)	5 (6.5)
M6	19 (6.7)	11 (5.7)	1 (6.2)	7 (9.1)
M7	3 (1.1)	2 (1.0)	0 (0.0)	1 (1.3)
Other	42 (14.7)	2 (1.0)	0 (0.0)	2 (2.6)
Unknown	4 (1.4)	34 (17.7)	2 (12.5)	6 (7.8)
Cytogenetic risk				
Favorable	36 (12.6)	25 (13.0)	1 (6.2)	10 (13.0)
Intermediate	125 (43.9)	87 (45.3)	10 (62.5)	28 (36.4)
Poor	114 (40.0)	73 (38.0)	5 (31.2)	36 (46.8)
Unevaluable	10 (3.5)	7 (3.6)	0 (0.0)	3 (3.9)
Disease risk at transplantation				
Low risk (CR1 & CR2)	141 (49.5)	97 (50.5)	7 (43.8)	37 (48.1)
High risk (CR3- & NR)	144 (50.5)	95 (49.5)	9 (56.2)	40 (51.9)
Donor Source				
Rel-BM	13 (4.6)	11 (5.7)	1 (6.2)	1 (1.3)
Rel-PB	47 (16.5)	32 (16.7)	1 (6.2)	14 (18.2)
UR-BM	86 (30.2)	55 (28.6)	8 (50.0)	23 (29.9)
UR-PB	11 (3.9)	8 (4.2)	0 (0.0)	3 (3.9)
UR-CB	128 (44.9)	86 (44.8)	6 (37.5)	36 (46.8)
Conditioning				
Myeloablative	98 (34.4)	65 (33.9)	9 (56.2)	24 (31.2)
Reduced intensity	187 (65.6)	127 (66.1)	7 (43.8)	53 (68.8)
Primary malignancy				
Breast cancer	75 (26.3)	32 (16.7)	8 (50.0)	35 (45.5)
Gynecological cancer	32 (11.2)	25 (13.0)	1 (6.2)	6 (7.8)
Gastroenterological cancer	23 (8.1)	16 (8.3)	0 (0.0)	7 (9.1)
Germ cell tumor	16 (5.6)	10 (5.2)	2 (12.5)	4 (5.2)
Malignant lymphoma	72 (25.3)	60 (31.2)	0 (0.0)	12 (15.6)
Acute leukemia	29 (10.2)	28 (14.6)	0 (0.0)	1 (1.3)
Other	38 (13.3)	21 (10.9)	5 (31.2)	12 (15.6)
Chemotherapy to primary malignancy				
Alkylator based	65 (22.8)	45 (23.4)	-	20 (26.0)
Topo II inhibitor based	24 (8.4)	23 (12.0)	-	1 (1.3)
Alkylator + topo II inhibitor based	158 (55.5)	106 (55.2)	-	52 (67.5)
Other	22 (7.7)	18 (9.4)	-	4 (5.2)
No chemotherapy (radiation alone)	16 (5.6)	-	16 (100)	-
Autologous PBSCT to primary malignancy				
No	260 (91.2)	169 (88.0)	16 (100.0)	75 (97.4)
Yes	25 (8.8)	23 (12.0)	0 (0.0)	2 (2.6)
Time from therapy for primary malignancy to diagnosis of t-AML (years, median [range])	4.5 [0.1–24.4]	4.0 [0.1–24.4]	3.7 [0.3–13.0]	5.1 [0.7–20.7]
Median follow-up of survivors (years, median [range])	3.4 [0.1–10.0]	3.6 [0.1–10.0]	2.8 [1.5–4.8]	3.0 [0.2–8.0]

Abbreviations: *CB* cord blood, *CHT* chemotherapy, *CR* complete remission, *ECOG-PS* Eastern Cooperative Oncology Group Performance Status, *FAB* French–American–British, *HCT-CI* hematopoietic cell transplantation-specific comorbidity index, *NR* non-remission, *PBSCT* peripheral blood stem cell transplantation, *Rel-BM* related bone marrow, *Rel-PB* related peripheral blood, *RT* radiation therapy, *t-AML* therapy-related acute myeloid leukemia, *topo* topoisomerase, *UR-BM* unrelated bone marrow, *UR-PB* unrelated peripheral blood

### Transplant outcomes of t-AML

**Fig. 1:**
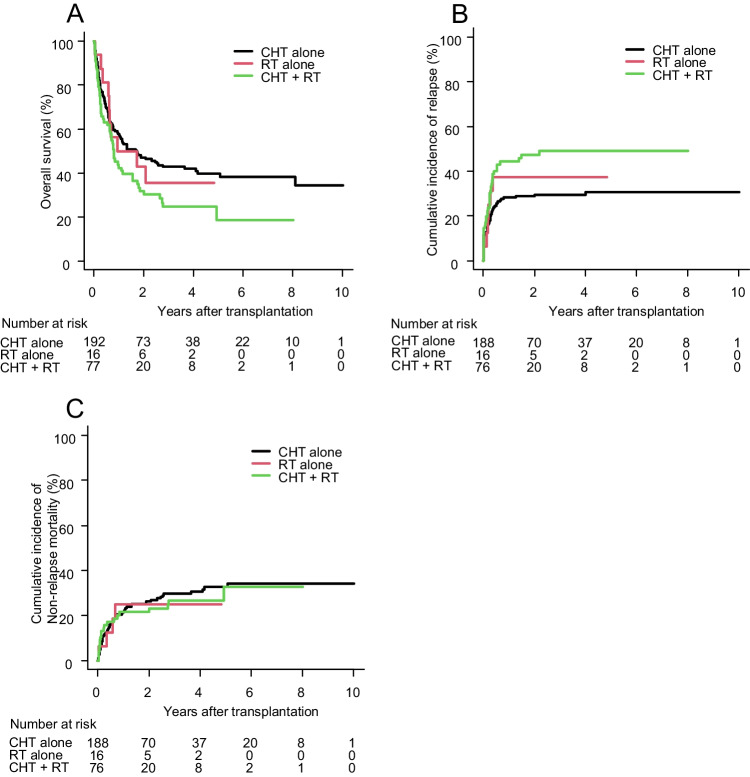
Comparison of allo-HSCT outcome according to type of treatment for t-AML. Overall survival (**A**), relapse incidence (**C**), and non-relapse mortality (**D**). Abbreviation: allo-HSCT allogeneic hematopoietic stem cell transplantation, CHT chemotherapy, RT radiation therapy, t-AML therapy-related acute myeloid leukemia

The 3-year OS for t-AML patients was 37.5% (95% CI: 31.6–43.5%) and the median duration was 13.8 months (95% CI: 9.5–21.7) (Supplemental Figure 1A). In univariate analysis regarding factors associated with t-AML, the type of primary malignancy, type of chemotherapy agent, and history of auto-PBSCT were not significant, whereas patients who received CHT + RT as a treatment for primary malignancy had significantly worse OS than those who received CHT alone (hazard ratio (HR) 1.51; 95% CI: 1.09–2.09; *p* = 0.014) (Table 2). The median OS with CHT alone, RT alone, and CHT + RT was 20.8, 15.9, and 9.2 months, respectively (*p* = 0.045) (Fig. 1A). The multivariable analysis showed that history of CHT + RT was an independent poor prognostic factor for OS (HR 1.65; 95% CI: 1.13–2.39; *p* = 0.009), along with male sex, PS 2-4, and high disease risk (Table 2).

**Table 2:** Univariable and multivariable analyses for OS, RI, and NRM in t-AML patients

No. of patients, *N* (%)	285(100)	OS				RI				NRM			
		Univariable	*P*	Multivariable	*P*	Univariable	*P*	Multivariable	*P*	Univariable	*P*	Multivariable	*P*
		HR (95% CI)		HR (95% CI)		HR (95% CI)		HR (95% CI)		HR (95% CI)		HR (95% CI)	
Age													
16-54	120 (42.1)	Reference		Reference						Reference		Reference	
≧ 55	165 (57.9)	1.47 (1.08–2)	0.015	1.24 (0.89–1.74)	0.21					2.12 (1.31–3.41)	0.002	1.89 (1.15–3.12)	0.013
Sex													
Female	166 (58.2)	Reference		Reference						Reference		Reference	
Male	119 (41.8)	1.72 (1.27–2.32)	< 0.001	1.63 (1.16–2.29)	0.004					1.54 (1–2.38)	0.052	1.51 (0.95–2.39)	0.078
ECOG-PS													
0-1	267 (93.7)	Reference		Reference						Reference		Reference	
2-4	18 (6.3)	3.95 (2.38–6.55)	< 0.001	2.77 (1.61–4.79)	< 0.001					1.64 (0.7–3.86)	0.26	1.50 (0.63–3.56)	0.36
HCT-CI													
0	52 (18.2)	Reference		Reference						Reference		Reference	
1-2	20 (7.0)	1.52 (0.79–2.90)	0.21	1.32 (0.67–2.62)	0.42					1.71 (0.72–4.1)	0.23	1.72 (0.69–4.3)	0.25
3-	208 (73.0)	1.06 (0.71–1.56)	0.79	0.99 (0.65–1.51)	0.96					1.07 (0.59–1.92)	0.83	1.20 (0.65–2.21)	0.56
Cytogenetic risk													
Favorable	36 (12.6)	Reference		Reference		Reference		Reference					
Intermediate	125 (43.9)	1.32 (0.77–2.29)	0.31	1.11 (0.63–1.96)	0.72	1.59 (0.68–3.72)	0.29	1.38 (0.55–3.42)	0.49				
Poor	114 (40.0)	2.38 (1.39–4.08)	0.001	1.27 (0.71–2.27)	0.42	3.61 (1.59–8.19)	0.002	2.33 (0.96–5.64)	0.061				
Disease risk at transplantation													
Low risk (CR1 & CR2)	141 (49.5)	Reference		Reference		Reference		Reference					
High risk (CR3 & NR)	144 (50.5)	3.37 (2.45–4.65)	< 0.001	2.97 (2.04–4.31)	< 0.001	3.15 (2.07–4.79)	< 0.001	3.10 (1.96–4.91)	< 0.001				
Donor source													
Rel-BM	13 (4.6)	Reference		Reference		Reference		Reference		Reference		Reference	
Rel-PB	47 (16.5)	2.84 (1.00–8.12)	0.051	1.97 (0.67–5.83)	0.22	1.85 (0.55–6.23)	0.32	1.17 (0.3–4.54)	0.82	1.75 (0.43–7.15)	0.43	1.56 (0.44–5.57)	0.49
UR-BM	86 (30.2)	3.05 (1.10–8.44)	0.031	2.41 (0.85–6.84)	0.098	1.53 (0.46–5.07)	0.49	1.01 (0.27–3.86)	0.98	2.51 (0.66–9.59)	0.18	2.12 (0.64–7)	0.22
UR-PB	11 (3.9)	2.03 (0.51–8.14)	0.32	1.03 (0.23–4.69)	0.97	3.26 (0.89–11.89)	0.074	1.42 (0.34–5.9)	0.63	0.00 (0–0)	0	0.00 (0–0)	0
UR-CB	128 (44.9)	3.54 (1.30–9.67)	0.014	1.99 (0.71–5.61)	0.19	1.56 (0.48–5.06)	0.46	0.74 (0.2–2.78)	0.66	2.81 (0.75–10.51)	0.12	2.15 (0.67–6.85)	0.2
Conditioning													
Myeloablative	98 (34.4)	Reference		Reference		Reference		Reference		Reference		Reference	
Reduced intensity	187 (65.6)	1.12 (0.81–1.53)	0.5	1.28 (0.91–1.81)	0.16	1.27 (0.83–1.95)	0.27	1.41 (0.9–2.21)	0.13	1.01 (0.64–1.6)	0.97	0.96 (0.59–1.55)	0.86
Type of primary malignancy													
Solid tumor	178 (62.5)	Reference				Reference				Reference			
Hematological malignancy	107 (37.5)	0.97 (0.72–1.33)	0.87			0.94 (0.63–1.4)	0.76			0.92 (0.58–1.46)	0.73		
Treatment for primary malignancy													
Chemotherapy alone	192 (67.4)	Reference		Reference		Reference		Reference		Reference			
Radiation alone	16 (5.6)	1.10 (0.57–2.10)	0.78	1.17 (0.59–2.31)	0.66	1.33 (0.58–3.04)	0.5	1.42 (0.59–3.41)	0.44	0.84 (0.3–2.32)	0.73		
Chemotherapy + radiation	77 (27.0)	1.51 (1.09–2.09)	0.014	1.65 (1.13–2.39)	0.009	1.79 (1.19–2.68)	0.004	1.62 (1.05–2.5)	0.029	0.89 (0.53–1.49)	0.66		
Chemotherapy for primary malignancy													
Alkylator based	65 (22.8)	Reference				Reference				Reference			
Topo II inhibitor based	24 (8.4)	0.50 (0.24–1.03)	0.06			0.72 (0.29–1.79)	0.48			0.49 (0.17–1.39)	0.18		
Alkylator + topo II inhibitor based	158 (55.5)	1.11 (0.77–1.59)	0.59			1.18 (0.73–1.9)	0.49			0.96 (0.57–1.61)	0.87		
Other	22 (7.7)	0.63 (0.33–1.24)	0.18			0.48 (0.17–1.39)	0.18			1.07 (0.51–2.27)	0.86		
No chemotherapy (radiation alone)	16 (5.6)	0.94 (0.47–1.87)	0.86			1.13 (0.47–2.74)	0.78			0.80 (0.27–2.36)	0.69		
Autologous PBSCT for primary malignancy													
No	260 (91.2)	Reference				Reference				Reference			
Yes	25 (8.8)	0.95 (0.56–1.61)	0.84			0.73 (0.36–1.48)	0.38			1.38 (0.68–2.8)	0.37		

Abbreviations: *NRM* non-relapse mortality, *OS* overall survival, *RI* relapse incidence. Other abbreviations are explained in Table 1

The 3-year RI for t-AML patients was 35.2% (95% CI: 29.6–40.9%) (Supplemental Figure 1B). In univariate analysis, patients with prior CHT + RT had significantly increased RI compared to CHT alone (HR 1.79; 95% CI: 1.19–2.68; *p* = 0.004) (Table 2). The 3-year RI for CHT alone, RT alone, and CHT + RT was 29.6%, 37.5%, and 49.0%, respectively (*p* = 0.019) (Fig. 1B). The multivariable analysis showed that high disease risk and prior CHT + RT were independent poor prognostic factors for RI (HR 3.10; 95% CI: 1.96–4.91; *p* < 0.001 and HR 1.62; 95% CI: 1.05–2.5; *p* = 0.029, respectively), whereas cytogenetic risk was not significant (HR 2.33; 95% CI: 0.96–5.64; *p* = 0.061) (Table 2). The 3-year NRM for t-AML patients was 28.6% (95% CI: 23.3–34.2%) (Supplemental Figure 1C). Factors associated with t-AML, including the type of primary malignancy, treatment, chemotherapy agent, and prior auto-PBSCT, were not significant for NRM in univariate analysis (Table 2). The 3-year NRM for CHT alone, RT alone, and CHT + RT was 29.7%, 25.0%, and 26.7%, respectively (*p* = 0.90) (Fig. 1C).

**Fig. 2:**
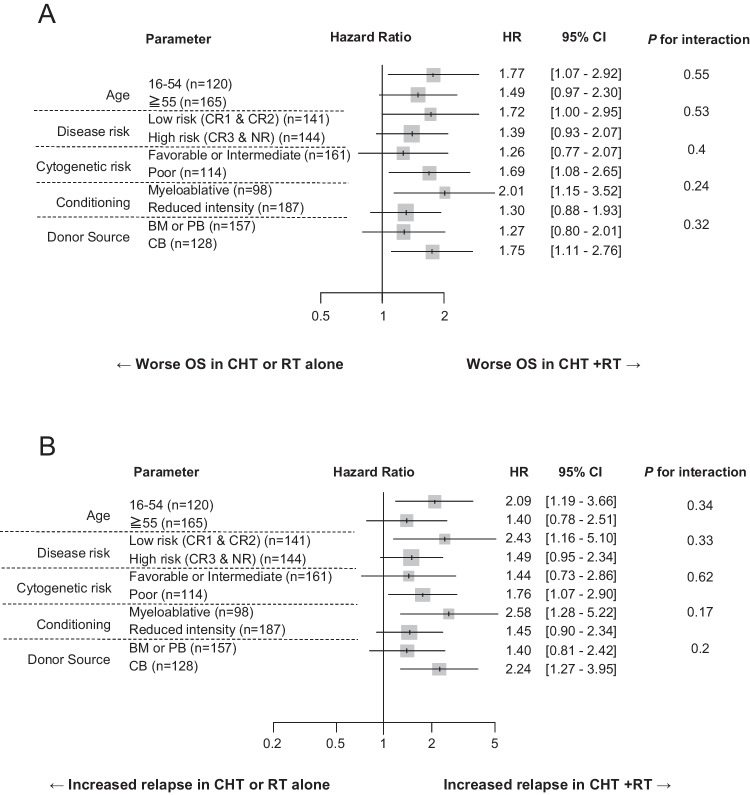
Hazard ratio of t-AML with prior CHT + RT for overall survival and relapse incidence in each subgroup. Overall survival (**A**) and relapse incidence (**B**). Abbreviation: Abbreviations: BM bone marrow, CB cord blood, CHT chemotherapy, CR complete remission, HR hazard ratio, NR non-remission, OS overall survival, PB peripheral blood, RT radiation therapy, t-AML therapy-related acute myeloid leukemia

Regarding patient background, the CHT + RT group had younger (*p* = 0.007) patients with more HCT-CI ≥ 3 values (*p* = 0.002) and less frequent hematological malignancies as the primary malignancy (*p* < 0.001) than CHT or RT alone group, whereas other variables were not significantly different (Table 1). The subgroup analysis was as follows: age (16–50 and ≥ 51 years), disease risk (CR1 & CR2 and CR3- & NR), cytogenetic risk (favorable or intermediate and poor), conditioning (MAC and RIC), and donor source (BM or PB and CB). All the HRs for OS and RI tended to be worse in the CHT + RT group. These subgroups had no interaction with the type of treatment for primary malignancy (Fig. 2).

Compared to CHT alone, cytogenetic risk and disease risk did not differ significantly in the CHT + RT group (*p* = 0.34 and *p* = 0.78, respectively). However, in the poor cytogenetic risk group, complex karyotype was significantly increased in the CHT + RT group (*p* = 0.007), whereas − 5/5q, − 7/7q, -17/17p, and monosomal karyotype were not significantly different (Supplemental Table 3).

### Comparison of patient characteristics and transplant outcome between t-AML and de novo AML

A total of 6761 patients with de novo AML were identified from the registry data. The comparison of patient characteristics between de novo AML and t-AML is presented in Supplemental Table 4. Older age, female sex, PS 2-4, HCT-CI ≥ 3, poor cytogenetic risk, and high disease risk were significantly more frequent in the t-AML group than the de novo AML group. Comparing between de novo AML and t-AML overall, the 3-year OS, DFS, RI, and NRM were significantly worse in t-AML (OS: 51.0% vs. 37.5%, *p* < 0.001; DFS: 46.9% vs. 36.2%, *p* < 0.001; RI: 31.0% vs. 35.2%, *p* = 0.047; NRM: 22.1% vs. 28.6%, *p* = 0.0086, respectively) (Supplemental Figure 1A, B, C, D). The cumulative incidence of grade 2–4 and grade 3–4 acute GVHD at day 100 was not significantly different between de novo AML and t-AML (34.5% vs. 30.6%, *p* = 0.22 and 10.8% vs. 9.1%, *p* = 0.41, respectively), while chronic GVHD at the 3 years was significantly higher in de novo AML than in t-AML (29% vs. 21.6%, *p* = 0.01) (Supplemental Figure 1E, F, G). In univariate analysis, OS and RI were significantly worse in t-AML with CHT + RT than in de novo AML (Table 3). In multivariable analysis, t-AML with CHT + RT was an independent poor prognostic factor for OS (HR 1.44; 95% CI: 1.09–1.91; *p* = 0.011) and RI (HR 1.44; 95% CI: 1.01–2.06; *p* = 0.042) compared to de novo AML (Table 3).

**Table 3:** Univariable and multivariable analyses for OS, Relapse, and NRM in t-AML and de novo AML patients

No. of patients, *N* (%)	7046 (100)	OS				RI				NRM			
		Univariable	*P*	Multivariable	*P*	Univariable	*P*	Multivariable	*P*	Univariable	*P*	Multivariable	*P*
		HR (95% CI)		HR (95% CI)		HR (95% CI)		HR (95% CI)		HR (95% CI)		HR (95% CI)	
Age													
16–50	3430 (48.7)	Reference		Reference						Reference		Reference	
≧ 51	3616 (51.3)	1.71 (1.59–1.83)	< 0.001	1.50 (1.39–1.61)	< 0.001					2.13 (1.91–2.37)	< 0.001	1.93 (1.73–2.16)	< 0.001
Sex													
Female	2978 (42.3)	Reference		Reference						Reference		Reference	
Male	4068 (57.7)	1.21 (1.13–1.30)	< 0.001	1.19 (1.11–1.28)	< 0.001					1.25 (1.12–1.38)	< 0.001	1.22 (1.1–1.36)	< 0.001
ECOG-PS													
0–1	6852 (97.3)	Reference		Reference						Reference		Reference	
2–4	187 (2.7)	3.71 (3.16–4.35)	< 0.001	2.24 (1.89–2.65)	< 0.001					2.14 (1.64–2.78)	< 0.001	1.73 (1.31–2.29)	< 0.001
HCT-CI													
0	3993 (56.7)	Reference		Reference						Reference		Reference	
1–2	1832 (26.0)	1.29 (1.19–1.40)	< 0.001	1.13 (1.04–1.22)	0.004					1.27 (1.13–1.43)	< 0.001	1.15 (1.02–1.3)	0.021
3-	1153 (16.4)	1.62 (1.48–1.78)	< 0.001	1.18 (1.07–1.30)	< 0.001					1.66 (1.46–1.89)	< 0.001	1.36 (1.18–1.57)	< 0.001
Cytogenetic risk													
Favorable	917 (13.0)	Reference		Reference		Reference		Reference					
Intermediate	4280 (60.7)	1.41 (1.25–1.58)	< 0.001	1.38 (1.22–1.55)	< 0.001	1.37 (1.17–1.6)	< 0.001	1.35 (1.15–1.57)	< 0.001				
Poor	1421 (20.2)	2.75 (2.42–3.13)	< 0.001	2.27 (1.99–2.58)	< 0.001	2.99 (2.55–3.52)	< 0.001	2.43 (2.06–2.87)	< 0.001				
Unevaluable	428 (6.1)	1.62 (1.35–1.93)	< 0.001	1.57 (1.31–1.88)	< 0.001	1.60 (1.27–2)	< 0.001	1.53 (1.21–1.92)	< 0.001				
Disease risk at transplantation													
Low risk (CR1 & CR2)	4207 (59.7)	Reference		Reference		Reference		Reference					
High risk (CR3 & NR)	2838 (40.3)	2.87 (2.68–3.07)	< 0.001	2.46 (2.29–2.65)	< 0.001	3.37 (3.09–3.68)	< 0.001	3.24 (2.96–3.55)	< 0.001				
Donor source													
Rel-BM	483 (6.9)	Reference		Reference		Reference		Reference		Reference		Reference	
Rel-PB	1651 (23.4)	1.63 (1.39–1.91)	< 0.001	1.27 (1.08–1.49)	0.004	1.37 (1.15–1.64)	< 0.001	1.01 (0.84–1.21)	0.94	1.52 (1.16–1.97)	0.002	1.45 (1.1–1.89)	0.007
UR-BM	2298 (32.6)	1.26 (1.08–1.48)	0.003	1.14 (0.98–1.34)	0.097	0.87 (0.73–1.04)	0.14	0.80 (0.67–0.96)	0.015	1.73 (1.34–2.23)	< 0.001	1.61 (1.24–2.09)	< 0.001
UR-PB	185 (2.6)	1.03 (0.77–1.39)	0.82	0.90 (0.67–1.21)	0.5	0.90 (0.65–1.24)	0.52	0.81 (0.59–1.12)	0.21	1.21 (0.78–1.9)	0.4	1.04 (0.66–1.63)	0.87
UR-CB	2424 (34.4)	1.74 (1.49–2.04)	< 0.001	1.16 (0.99–1.36)	0.066	0.99 (0.83–1.19)	0.95	0.66 (0.55–0.79)	< 0.001	2.22 (1.72–2.86)	< 0.001	1.85 (1.43–2.4)	< 0.001
Conditioning													
Myeloablative	2336 (33.2)	Reference		Reference		Reference		Reference		Reference		Reference	
Reduced intensity	4710 (66.8)	1.06 (0.98–1.14)	0.14	1.12 (1.04–1.21)	0.002	1.12 (1.02–1.23)	0.017	1.18 (1.08–1.3)	< 0.001	0.96 (0.86–1.07)	0.48	0.95 (0.85–1.06)	0.37
Treatment for primary malignancy													
de novo AML	6761 (96.0)	Reference		Reference		Reference		Reference		Reference		Reference	
t-AML (chemotherapy alone)	192 (2.7)	1.27 (1.05–1.55)	0.013	1.00 (0.82–1.22)	0.99	1.01 (0.77–1.32)	0.96	0.85 (0.64–1.11)	0.24	1.43 (1.1–1.86)	0.008	1.08 (0.81–1.43)	0.62
t-AML (radiation alone)	16 (0.2)	1.41 (0.76–2.62)	0.28	1.03 (0.55–1.93)	0.92	1.37 (0.59–3.18)	0.46	1.12 (0.46–2.75)	0.8	1.20 (0.44–3.27)	0.73	0.95 (0.36–2.48)	0.91
t-AML (chemo + RT)	77 (1.1)	1.97 (1.51–2.57)	< 0.001	1.44 (1.09–1.91)	0.011	1.88 (1.34–2.62)	< 0.001	1.44 (1.01–2.06)	0.042	1.29 (0.82–2.03)	0.27	1.08 (0.68–1.72)	0.75

Abbreviations are explained in Tables 1 and 2

**Fig. 3:**
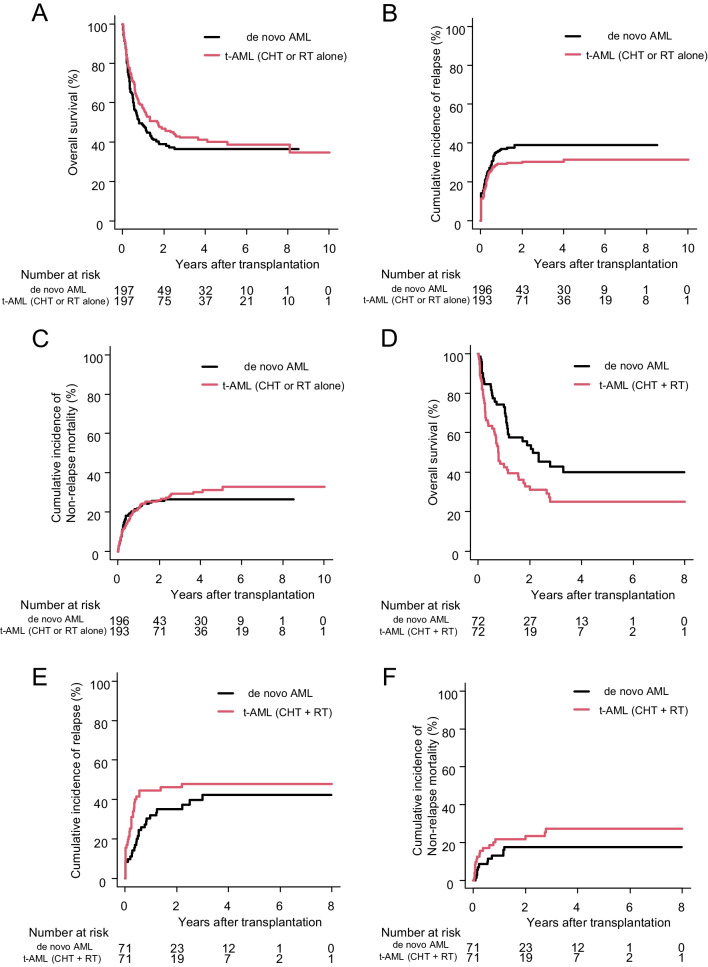
Comparison of transplant outcomes between t-AML and de novo AML in a propensity score-matched cohort. Overall survival (**A**), relapse incidence (**B**), and non-relapse mortality (**C**) with CHT or RT alone group of t-AML compared to de novo AML. Overall survival (**D**), relapse incidence (**E**), and non-relapse mortality (**F**) with CHT and RT group of t-AML compared to de novo AML. Abbreviation: Abbreviations: AML acute myeloid leukemia, CHT chemotherapy, RT radiation therapy, t-AML therapy-related acute myeloid leukemia

Because several patient factors were significantly different between the t-AML and de novo AML groups, we also performed a PSM analysis in t-AML with CHT or RT alone vs. de novo AML, and t-AML with CHT + RT vs. de novo AML to confirm the results of the multivariable Cox model. The covariates included were age, sex (male vs. female), disease risk at transplantation (low risk vs. high risk), HCT-CI (0 vs. 1–2 vs. 3-), donor source (Rel-BM vs. Rel-PB vs. UR-BM vs. UR-PB vs. CBT), conditioning (MAC vs. RIC), PS (0–1 vs. 2–4), and cytogenetic risk (favorable vs. intermediate vs. poor). In the final PSM cohort, 197 patients were assigned to each de novo AML and t-AML with CHT or RT alone group (Supplemental Table 5), and 72 patients were assigned to each de novo AML and t-AML with the CHT + RT group (Supplemental Table 6), respectively. Patient backgrounds were well balanced (Supplemental Figure 2) with no significant difference between the groups in each final cohort. The 3-year OS did not differ significantly between the de novo AML and t-AML with CHT or RT alone groups (36.4%; 95% CI: 29.2–43.6 and 42.3%; 95% CI: 34.9–49.3, *p* = 0.16). RI and NRM were also not significantly different (Fig. 3A, B, C). However, compared with de novo AML, t-AML with CHT + RT tended to increase RI and NRM with significantly poor prognosis in 3-year OS (42.7%; 95% CI: 29.7–55.2 and 25.2%; 95% CI: 15.1–36.5, *p* = 0.009) (Fig. 3D, E, F).

## Discussion

In this large cohort study, we obtained data for 285 t-AML patients who underwent allo-HSCT using an additional nationwide survey. This analysis revealed that t-AML patients who received CHT + RT as therapy for primary malignancy were associated with dismal outcomes after allo-HSCT. Compared to t-AML with prior CHT or RT alone and de novo AML, t-AML with CHT + RT revealed increased relapse and significantly worse OS. In the PSM cohort, t-AML with CHT + RT resulted in worse OS than de novo AML.

According to recent retrospective studies, t-AML patients who underwent allo-HSCT were more likely to be female, elderly, have poor cytogenetic risk, and have a history of breast cancer and malignant lymphoma as primary malignancy [5, 24–26], and OS, RI, and NRM were 25.0–40.4%, 37.7–43.2%, and 25.6–33.6%, respectively [24–26]. These results were comparable with our present study, whereas this study population was more likely to have high disease risk (CR3- and NR) and received CB transplantation. Similar to the results of the present study, age [14–16], cytogenetic risk [14–16, 26], disease status at allo-HSCT [15, 16, 26], donor source [15, 26], performance status [16, 26], and conditioning [26] were reported as prognostic factors, and some studies show that the cumulative toxicity of therapy for primary malignancy may affect the NRM of allo-HSCT in t-AML patients [5, 27]. To the best of our knowledge, this is the first study to report that a history of CHT + RT for primary malignancy may lead to a worse prognosis of allo-HSCT. In our study, primary hematologic malignancies were predominantly treated with CHT alone. The use of RT in combination with CHT was less common compared to solid tumors such as breast cancer. This resulted in a lower percentage of hematologic malignancies and a higher percentage of solid tumors in the CHT + RT group. In addition, the HCT-CI incorporates “Prior solid tumor” as a factor that adds 3 points to the score. Consequently, the proportion of patients with an HCT-CI score of 3 or higher was also greater in the CHT + RT group. The CHT + RT group had a younger age profile compared to the CHT or RT group, likely due to a higher representation of breast cancer patients, who tend to be diagnosed at a younger age compared to patients with other primary malignancies. Interestingly, despite their younger age, the CHT + RT group had a worse transplant prognosis than the CHT or RT group. Even after adjusting for patient background for all the above factors, prior CHT + RT was still an independent poor prognostic factor for OS and RI.

In the pathogenesis of t-AML, distinct mechanisms were reported, such as direct induction of a fusion oncogene such as PML/RARA or MLL gene mediated by TI [28, 29], selective expansion of preexisting clonal hematopoiesis with TP53 mutation [30–32], and chemotherapy-induced alterations to the bone marrow microenvironment [33]. CHT + RT, which is a more intensive treatment than CHT or RT alone, may affect these pathological mechanisms, causing tumor resistance to conditioning and graft-versus-leukemia effect, which may worsen the clinical outcome.

In this study, the CHT + RT group had significantly more complex karyotypes than the CHT alone group. The prognosis for t-AML patients with complex karyotypes is poor [10], and it is often associated with TP53 mutation [34]. TP53 mutation is the most frequent mutation in t-AML that infers a dismal prognosis [30, 34]. In this study, patients with complex karyotypes may have a higher rate of TP53 mutation, which may explain increased relapse.

There are several limitations in the present study. This is a retrospective and registry database-based study. The missing data, uncollected information, and excluded cases might lead to residual confounding results, although we attempted to collect detailed information using the additional nationwide survey. In particular, no data on genomic abnormalities in t-AML were available. Although FLT3-ITD mutation was reported to be more infrequent in t-AML than in de novo AML [5, 35], only a small number of patients were evaluated, and the majority had missing data in this study (data not shown). Other genomic abnormalities such as TP53 mutation were not assessed. The lack of data on these genomic abnormalities may have resulted in bias in adjusting for patient background. In the present analysis, it is unclear how CHT + RT actually affects the pathomechanism of t-AML and increases relapse. The CHT + RT group is heterogeneous, and identification of the detailed combination of the chemotherapy agent, drug and irradiation dose, and treatment duration that affect the therapeutic resistance of t-AML requires a larger sample study, due to the vast number of CHT + RT combinations.

In conclusion, our analyses suggest that t-AML patients who received CHT + RT for primary malignancy were associated with increased relapse and worse OS in allo-HSCT. These results provide further evidence of the pathogenesis of t-AML and may contribute to a refined treatment strategy with allo-HSCT for t-AML.

## Supplementary information

ESM 1 (DOCX 163 kb)
