# Supplementary material for: Poor outcome of allogeneic transplantation for therapy-related acute myeloid leukemia induced by prior chemoradiotherapy
Source: Ann Hematol. 2023 Jul 21;102(10):2879–93. doi: 10.1007/s00277-023-05356-6 (PMC10492731; doi:10.1007/s00277-023-05356-6)
Supplement: Supplementary file 1 — (DOCX 163 kb) [file 277_2023_5356_MOESM1_ESM.docx]

**Supplemental Figure Legends**

**Supplemental Figure 1. Comparison of transplant outcomes between t-AML and de novo AML.** Overall survival (A), Disease free survival (B), Relapse incidence (C), Non-relapse mortality (D), acute GVHD (Grade 2-4) (E), acute GVHD (Grade 3-4) (F), and chronic GVHD (G). Abbreviations: GVHD, graft-versus-host disease; t-AML, therapy-related acute myeloid leukemia

**Supplemental Figure 2. Histogram of propensity scores in a matched-cohort.** t-AML with CHT or RT alone and de novo AML cohort (A), and t-AML with CHT + RT and de novo AML cohort (B). Abbreviations: AML, acute myeloid leukemia; CHT, chemotherapy; RT, radiation therapy; t-AML, therapy-related acute myeloid leukemia

**Supplemental Figure 1.**


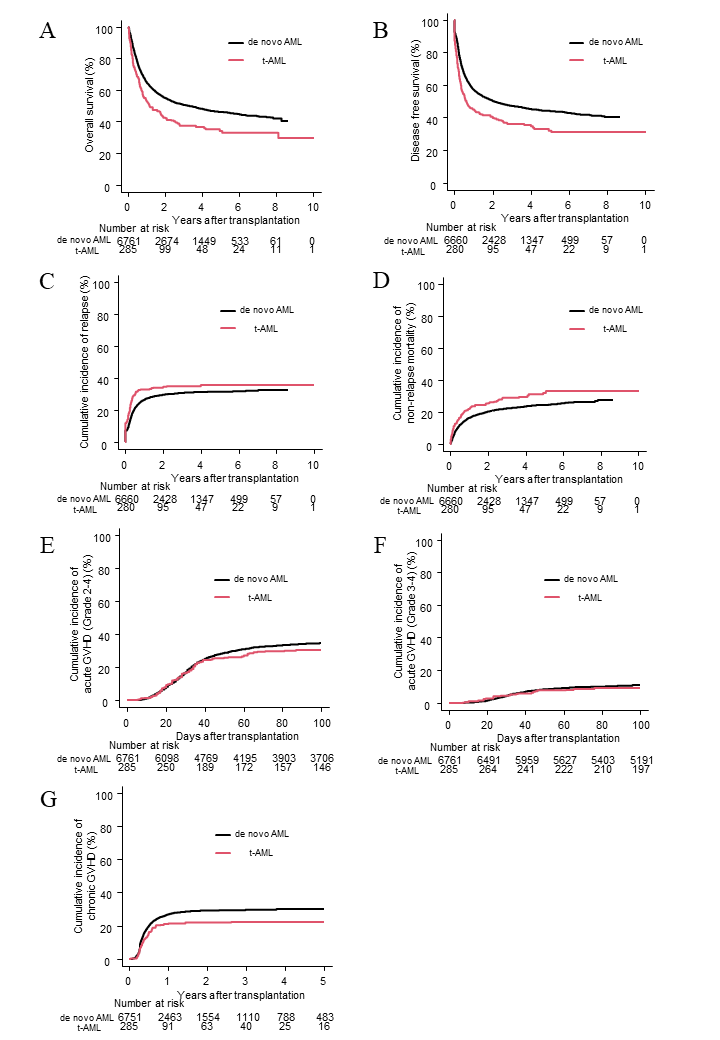


**Supplemental Figure 2.**


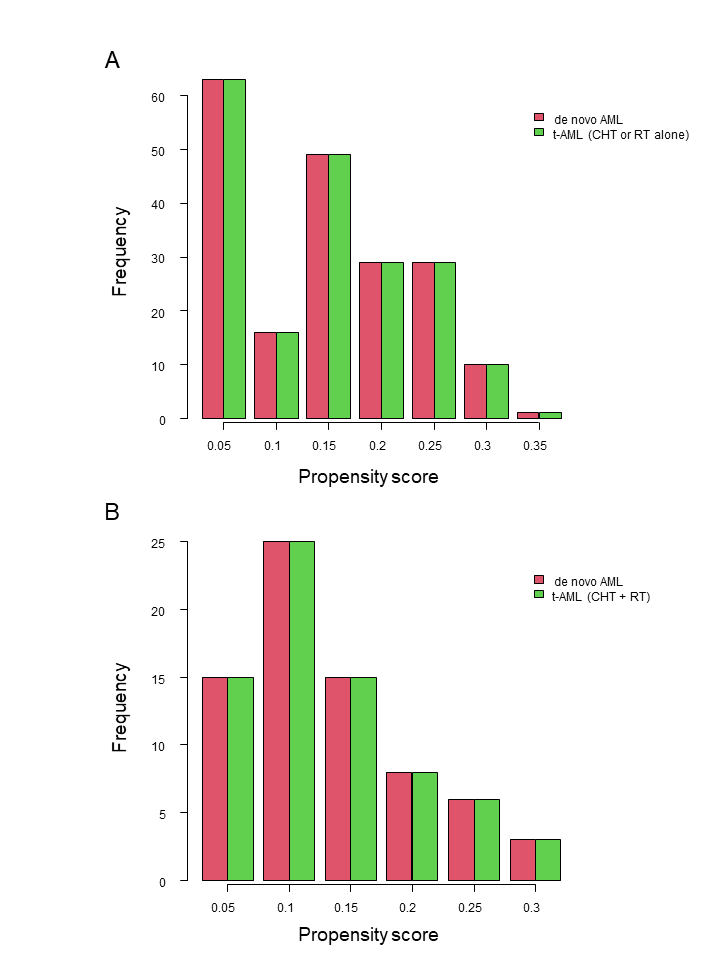


| **Supplemental Table 1. Characteristics of primary malignancy and t-AML** | | |
| --- | --- | --- |
| No. of patients, N (%) | 285(100) |  |
| Primary malignancy |  |  |
| Solid tumor | 178 (62.5) |  |
| Breast cancer |  | 75 |
| Gynecological cancer |  |  |
| Ovarian cancer |  | 17 |
| Endometrial cancer |  | 10 |
| Cervical cancer |  | 5 |
| Gastroenterological cancer |  |  |
| Colon cancer |  | 12 |
| Esophagus cancer |  | 10 |
| Stomach cancer |  | 1 |
| Germ cell tumor |  | 16 |
| Head and neck cancer |  | 7 |
| Bone and soft tissue sarcoma |  | 6 |
| Lung cancer |  | 4 |
| Malignant melanoma |  | 3 |
| Urinary organ cancer |  | 3 |
| Thyroid cancer |  | 2 |
| Hepatobiliary cancer |  | 2 |
| Brain tumor |  | 2 |
| Thymic carcinoma |  | 1 |
| Pancreatic cancer |  | 1 |
| Neuroendocrine neoplasm |  | 1 |
| Hematological malignancy | 107 (37.5) |  |
| Malignant lymphoma |  |  |
| Diffuse large B cell lymphoma |  | 25 |
| Follicular lymphoma |  | 20 |
| Burkitt lymphoma |  | 5 |
| Marginal zone lymphoma |  | 5 |
| Chronic lymphocytic leukemia |  | 2 |
| Mantle cell lymphoma |  | 2 |
| Primary mediastinal large B cell lymphoma | | 1 |
| Peripheral T-cell lymphoma |  | 5 |
| Hodgkin lymphoma |  | 1 |
| unknown |  | 6 |
| Plasma cell neoplasm |  | 5 |
| Acute leukemia |  |  |
| Acute lymphoblastic leukemia/lymphoma | | 10 |
| Acute myeloid leukemia (non-APL)* | | 10 |
| Acute promyelocytic leukemia |  | 9 |
| Myeloproliferative neoplasms** |  | 1 |
| Chemotherapy for t-AML |  |  |
| Intensive chemotherapy | 157(55.1) |  |
| Non-intensive chemotherapy | 34(11.9) |  |
| No chemotherapy (upfront allo-HSCT) | 5(1.8) |  |
| unknown | 89(31.2) |  |
| Time from diagnosis t-AML to allo-HSCT (days, median [range]) | 168 [9 - 3703] |  |

* One patient was treated for de novo AML (M5), diagnosed with t-MDS 506 days later, and progressed to t-AML. The timing of treatment initiation for de novo AML was unknown in three cases, but the diagnosis of de novo AML was at least 4.5 years before the diagnosis of t-AML, and FAB classification, surface markers, or cytogenetic abnormalities were different between de novo AML and t-AML. The remaining patients had at least 6 years between treatment of de novo AML and diagnosis of t-AML.

** Hydroxycarbamide was used for essential thrombocythemia from 16 to 8.5 years before the diagnosis of t-AML, but the detailed clinical course is unknown.

Abbreviations: allo-HSCT, allogeneic hematopoietic stem cell transplantation; AML, acute myeloid leukemia; APL, acute promyelocytic leukemia; t-AML, therapy-related acute myeloid leukemia; t-MDS, therapy-related myelodysplastic syndrome

| Supplemental Table 2. Radiation site and dose to the primary malignancy in t-AML with CHT + RT. |  |
| --- | --- |
| No. of patients, N | 77 |
| Primary malignancy / Radiation site / Radiation dose |  |
| Solid tumor |  |
| Breast cancer |  |
| Primary lesion / 50 - 60 Gy | 24 |
| Primary lesion / unknown | 3 |
| Cervical spine to lumbar spine / unknown | 1 |
| unknown / unknown | 7 |
| Cervical cancer |  |
| Primary lesion + vaginal irradiation / 50.4 Gy + 17 Gy | 1 |
| Primary lesion + Intra-abdominal lymph node lesions / 30 Gy + 45 Gy | 1 |
| unknown / unknown | 1 |
| Endometrial cancer |  |
| Primary lesion / 54 Gy | 1 |
| Pelvic cavity lesion / 50 Gy | 1 |
| Ovarian cancer |  |
| unknown / unknown | 1 |
| Esophagus cancer |  |
| Primary lesion / 50 - 60 Gy | 4 |
| unknown / unknown | 2 |
| Colon cancer |  |
| Primary lesion / 40 Gy | 1 |
| Germ cell tumor |  |
| Primary lesion (intracranial lesions) / 30.6 Gy | 1 |
| Primary lesion (pelvic cavity + renal portal lesion) / 25 Gy + 10 Gy | 1 |
| Mediastinal metastases / 60 Gy | 1 |
| unknown / unknown | 1 |
| Head and neck cancer |  |
| Primary lesion / 66 Gy | 2 |
| Bone and soft tissue sarcoma |  |
| Primary lesion / 36 Gy | 1 |
| Primary lesion / unknown | 2 |
| Intra-abdominal lymph node lesions / 50 Gy | 1 |
| unknown / unknown | 2 |
| Brain tumor |  |
| Primary lesion / 50 - 54 Gy | 2 |
| Neuroendocrine neoplasm |  |
| Intra-abdominal lesions / 30 Gy | 1 |
|  |  |
| Hematological malignancy |  |
| Diffuse large B cell lymphoma |  |
| Head / 16.5 Gy | 1 |
| Pharynx / 50 Gy | 1 |
| Stomach / 40 Gy | 1 |
| unknown / unknown | 2 |
| Follicular lymphoma |  |
| Para-aortic lymph nodes / 18 Gy | 1 |
| Cervical lymph nodes / 40 Gy | 1 |
| Inguinal lymph nodes / 36 Gy | 1 |
| Retroperitoneum, parotid gland, axilla, and inguinal / total 70 Gy | 1 |
| Retroperitoneum / 40 Gy | 1 |
| Marginal zone lymphoma |  |
| Lung / 4 Gy | 1 |
| unknown / unknown | 1 |
| Plasma cell neoplasm |  |
| Right ribs, neck, and right forearm / 36Gy, 37.5Gy, and 36Gy | 1 |
| Acute lymphoblastic leukemia/lymphoma |  |
| unknown / unknown | 1 |

Abbreviations: CHT, chemotherapy; RT, radiation therapy; t-AML, therapy-related acute myeloid leukemia

**Supplemental Table ~~2~~3. Type of poor cytogenetic risk in t-AML patients.**

|  |  |  |  |
| --- | --- | --- | --- |
|  | CHT alone | CHT +RT |  |
| No. of patients, N (%) | 73(100) | 36(100) | *P* |
| -5/5q- |  |  |  |
| no | 41 (56.2) | 16 (44.4) | 0.53 |
| yes | 29 (39.7) | 18 (50.0) |  |
| unevaluable | 3 (4.1) | 2 (5.6) |  |
| -7/7q- |  |  |  |
| no | 30 (41.1) | 18 (50.0) | 0.23 |
| yes | 43 (58.9) | 17 (47.2) |  |
| unevaluable | 0 (0.0) | 1 (2.8) |  |
| -17/17p- |  |  |  |
| no | 53 (72.6) | 22 (61.1) | 0.34 |
| yes | 17 (23.3) | 13 (36.1) |  |
| unevaluable | 3 (4.1) | 1 (2.8) |  |
| Monosomal karyotype |  |  |  |
| no | 40 (54.8) | 18 (50.0) | 0.87 |
| yes | 30 (41.1) | 17 (47.2) |  |
| unevaluable | 3 (4.1) | 1 (2.8) |  |
| Complex karyotype |  |  |  |
| no | 28 (38.4) | 5 (13.9) | **0.007** |
| yes | 42 (57.5) | 31 (86.1) |  |
| unevaluable | 3 (4.1) | 0 (0.0) |  |

Abbreviations: CHT, chemotherapy; RT, radiation therapy; t-AML, therapy-related acute myeloid leukemia

**Supplemental Table ~~3~~4. Patient characteristics of de novo AML and t-AML.**

|  |  |  |  |
| --- | --- | --- | --- |
|  | de novo AML | t-AML |  |
| No. of patients, N (%) | 6761(100) | 285(100) | *p* |
| Age |  |  |  |
| median [range] | 51 [16-80] | 57 [16-80] | <0.001 |
| 16-50 | 3340 (49.4) | 90 (31.6) | <0.001 |
| ≧51 | 3421 (50.6) | 195 (68.4) |  |
| Sex |  |  |  |
| Female | 2812 (41.6) | 166 (58.2) | <0.001 |
| Male | 3949 (58.4) | 119 (41.8) |  |
| Performance status |  |  |  |
| 0-1 | 6585 (97.4) | 267 (93.7) | 0.002 |
| 2-4 | 169 (2.5) | 18 (6.3) |  |
| unknown | 7 (0.1) | 0 (0.0) |  |
| HCT-CI |  |  |  |
| 0 | 3941 (58.3) | 52 (18.2) | <0.001 |
| 1-2 | 1812 (26.8) | 20 (7.0) |  |
| 3- | 945 (14.0) | 208 (73.0) |  |
| unknown | 63 (0.9) | 5 (1.8) |  |
| FAB classification |  |  |  |
| M0 | 520 (7.7) | 21 (7.4) |  |
| M1 | 1073 (15.9) | 30 (10.5) |  |
| M2 | 2643 (39.1) | 102 (35.8) |  |
| M3 | 71 (1.1) | 7 (2.5) |  |
| M4 | 984 (14.6) | 36 (12.6) |  |
| M5 | 696 (10.3) | 21(7.4) |  |
| M6 | 342 (5.1) | 19 (6.7) |  |
| M7 | 96 (1.4) | 3 (1.1) |  |
| other | 321 (4.7) | 42 (14.7) |  |
| unknown | 15 (0.2) | 4 (1.4) |  |
| Cytogenetic risk |  |  |  |
| favorable | 881 (13.0) | 36 (12.6) | <0.001 |
| intermediate | 4154 (61.4) | 125 (43.9) |  |
| poor | 1307 (19.3) | 114 (40.0) |  |
| unevaluable | 419 (6.2) | 10 (3.5) |  |
| Disease risk at transplantation |  |  |  |
| Low risk (CR1 & CR2) | 4066 (60.1) | 141 (49.5) | 0.001 |
| High risk (CR3 & NR) | 2694 (39.8) | 144 (50.5) |  |
| unknown | 1 (0.0) | 0 (0.0) |  |
| Donor Source |  |  |  |
| Rel-BM | 470 (7.0) | 13 (4.6) |  |
| Rel-PB | 1604 (23.7) | 47 (16.5) |  |
| UR-BM | 2212 (32.7) | 86 (30.2) |  |
| UR-PB | 174 (2.6) | 11 (3.9) |  |
| UR-CB | 2296 (34.0) | 128 (44.9) |  |
| unknown | 5 (0.1) | 0 (0.0) |  |
| Conditioning |  |  |  |
| Myeloablative | 2238 (33.1) | 98 (34.4) | 0.65 |
| Reduced intensity | 4523 (66.9) | 187 (65.6) |  |
| Time from diagnosis to HCT, days (median [range]) | 168 [9-3703] | 200 [0-9003] | <0.001 |
| Median follow-up of survivors, years (median [range]) | 3.0 [0.1-8.6] | 3.4 [0.1-10.0] | 0.08 |

Abbreviations: AML, acute myeloid leukemia; CB, cord blood; CR, complete remission; ECOG-PS, Eastern Cooperative Oncology Group Performance Status; FAB, French–American–British; HCT-CI, hematopoietic cell transplantation specific comorbidity index; NR, non-remission; Rel-BM, related bone marrow; Rel-PB, related peripheral blood; RT, radiation therapy; t-AML, therapy-related acute myeloid leukemia; UR-BM, unrelated bone marrow; UR-PB, unrelated peripheral blood

**Supplemental Table 5. Propensity-score matched cohort in de novo AML and t-AML with CHT or RT alone to primary malignancy.**

|  |  |  |  |
| --- | --- | --- | --- |
|  | de novo AML | t-AML (CHT or RT alone) |  |
| No. of patients, N (%) | 197(100) | 197(100) | *p** |
| Age |  |  |  |
| 16-57 | 96 (48.7) | 95 (48.2) | 1 |
| ≧58 | 101 (51.3) | 102 (51.8) |  |
| Sex |  |  |  |
| Female | 98 (49.7) | 106 (53.8) | 0.4 |
| Male | 99 (50.3) | 91 (46.2) |  |
| Performance status |  |  |  |
| 0-1 | 179 (90.9) | 182 (92.4) | 0.68 |
| 2-4 | 18 (9.1) | 15 (7.6) |  |
| HCT-CI |  |  |  |
| 0 | 42 (21.3) | 43 (21.8) | 0.77** |
| 1-2 | 19 (9.6) | 18 (9.1) |  |
| 3- | 136 (69.0) | 136 (69.0) |  |
| Cytogenetic risk |  |  |  |
| favorable | 16 (8.1) | 26 (13.2) | 0.11 |
| intermediate | 110 (55.8) | 94 (47.7) |  |
| poor | 71 (36.0) | 77 (39.1) |  |
| Disease risk at transplantation |  |  |  |
| Low risk (CR1 & CR2) | 94 (47.7) | 100 (50.8) | 0.6 |
| High risk (CR3 & NR) | 103 (52.3) | 97 (49.2) |  |
| Donor Source |  |  |  |
| Rel-BM | 9 (4.6) | 12 (6.1) | 0.75 |
| Rel-PB | 34 (17.3) | 32 (16.2) |  |
| UR-BM | 58 (29.4) | 62 (31.5) |  |
| UR-PB | 12 (6.1) | 7 (3.6) |  |
| UR-CB | 84 (42.6) | 84 (42.6) |  |
| Conditioning |  |  |  |
| Myeloablative | 67 (34.0) | 72 (36.5) | 0.66 |
| Reduced intensity | 130 (66.0) | 125 (63.5) |  |
| *Mantel–Haenszel test |  |  |  |
| ** Comparison HCT-CI 0-1 with HCT-CI 2- | |  |  |

Abbreviations: AML, acute myeloid leukemia; CB, cord blood; CHT, chemotherapy; CR, complete remission; ECOG-PS, Eastern Cooperative Oncology Group Performance Status; HCT-CI, hematopoietic cell transplantation specific comorbidity index; NR, non-remission; Rel-BM, related bone marrow; Rel-PB, related peripheral blood; RT, radiation therapy; t-AML, therapy-related acute myeloid leukemia; UR-BM, unrelated bone marrow; UR-PB, unrelated peripheral blood

**Supplemental Table 6. Propensity-score matched cohort in de novo AML and t-AML with CHT + RT to primary malignancy.**

|  |  |  |  |
| --- | --- | --- | --- |
|  | de novo AML | t-AML (Chemo and RT) |  |
| No. of patients, N (%) | 72(100) | 72(100) | *p** |
| Age |  |  |  |
| 16-50 | 30 (41.7) | 30 (41.7) | 1 |
| ≧51 | 42 (58.3) | 42 (58.3) |  |
| Sex |  |  |  |
| Female | 51 (70.8) | 50 (69.4) | 1 |
| Male | 21 (29.2) | 22 (30.6) |  |
| Performance status |  |  |  |
| 0-1 | 72 (100.0) | 69 (95.8) | 0.24 |
| 2-4 | 0 (0.0) | 3 (4.2) |  |
| HCT-CI |  |  |  |
| 0 | 8 (11.1) | 8 (11.1) | 1** |
| 1-2 | 1 (1.4) | 1 (1.4) |  |
| 3- | 63 (87.5) | 63 (87.5) |  |
| Cytogenetic risk |  |  |  |
| favorable | 6 (8.3) | 10 (13.9) | 0.066 |
| intermediate | 37 (51.4) | 27 (37.5) |  |
| poor | 29 (40.3) | 35 (48.6) |  |
| Disease risk at transplantation |  |  |  |
| Low risk (CR1 & CR2) | 38 (52.8) | 34 (47.2) | 0.58 |
| High risk (CR3 & NR) | 34 (47.2) | 38 (52.8) |  |
| Donor Source |  |  |  |
| Rel-BM | 1 (1.4) | 1 (1.4) | 0.9 |
| Rel-PB | 16 (22.2) | 13 (18.1) |  |
| UR-BM | 19 (26.4) | 22 (30.6) |  |
| UR-PB | 2 (2.8) | 3 (4.2) |  |
| UR-CB | 34 (47.2) | 33 (45.8) |  |
| Conditioning |  |  |  |
| Myeloablative | 24 (33.3) | 23 (31.9) | 1 |
| Reduced intensity | 48 (66.7) | 49 (68.1) |  |
| *Mantel–Haenszel test |  |  |  |
| ** Comparison HCT-CI 0 with HCT-CI 1- | |  |  |

Abbreviations: AML, acute myeloid leukemia; CB, cord blood; CHT, chemotherapy; CR, complete remission; ECOG-PS, Eastern Cooperative Oncology Group Performance Status; HCT-CI, hematopoietic cell transplantation specific comorbidity index; NR, non-remission; Rel-BM, related bone marrow; Rel-PB, related peripheral blood; RT, radiation therapy; t-AML, therapy-related acute myeloid leukemia; UR-BM, unrelated bone marrow; UR-PB, unrelated peripheral blood
